# Gamma, Gaussian and logistic distribution models for airborne pollen grains and fungal spore season dynamics

**DOI:** 10.1007/s10453-014-9332-8

**Published:** 2014-03-18

**Authors:** I. Kasprzyk, A. Walanus

**Affiliations:** 1 Department of Environmental Biology, University of Rzeszów, Zelwerowicza 4, 35-959 Rzeszow, Poland; 2 Faculty of Geology, Geophysics and Environmental Protection, AGH University of Science and Technology, al. A. Mickiewicza 30, 30-059 Kraków, Poland

**Keywords:** Gamma distribution, Gaussian distribution, Logistic differential function, Aerobiology, Modelling, Pollen season

## Abstract

The characteristics of a pollen season, such as timing and magnitude, depend on a number of factors such as the biology of the plant and environmental conditions. The main aim of this study was to develop mathematical models that explain dynamics in atmospheric concentrations of pollen and fungal spores recorded in Rzeszów (SE Poland) in 2000–2002. Plant taxa with different characteristics in the timing, duration and curve of their pollen seasons, as well as several fungal taxa were selected for this analysis. Gaussian, gamma and logistic distribution models were examined, and their effectiveness in describing the occurrence of airborne pollen and fungal spores was compared. The Gaussian and differential logistic models were very good at describing pollen seasons with just one peak. These are typically for pollen types with just one dominant species in the flora and when the weather, in particular temperature, is stable during the pollination period. Based on *s* parameter of the Gaussian function, the dates of the main pollen season can be defined. In spite of the fact that seasonal curves are often characterised by positive skewness, the model based on the gamma distribution proved not to be very effective.

## Introduction

The characteristics of a pollen season, such as timing, curve and magnitude, depend on a number of factors such as the biology of the plant, species spectrum and environmental conditions. Patterns in the occurrence of airborne pollen are largely chaotic, but some features of pollen seasons of specific taxa are characterised by relatively low variability (Wołek and Myszkowska 2008). A similar opinion was presented by Hyde (1956) who stressed that for many pollen taxa, it is possible to determine a “dense” period during which the pollen is present in the air at its maximum concentrations every year, e.g. for *Ulmus* or *Fagus*. Time series of pollen seasons from the same taxon in successive years can follow a different pattern (Comtois and Sherknies 1991; Piotrowska and Kubik-Komar 2012). This phenomenon is more noticeable in taxa with lower airborne pollen concentrations such as, for example, *Rumex.* This simply results from the statistics of pollen counts.

Moving averages, with varying degrees of smoothing, can be used to represent the curve of the pollen season. In the case of many taxa, this method does not always produce the expected result and it is still difficult to discern any trends or the characteristics of the season. When describing Urticaceae pollen time series, Belmonte and Canela (2002) applied a nonparametric method, Friedman’s super smoother. This method chooses from among three smoothers by cross-validation for the best prediction.

Mathematical models describing pollen season curves have also been presented in the literature. They are a greater or lesser simplification of the reality, but they provide a better understanding and interpretation of aerobiological phenomena and allow these phenomena to be predicted. The occurrence of airborne pollen/fungal spores has a seasonal nature, and the curves representing their temporal concentrations often have a shape that is close to a bell curve. A model based on a Gaussian function has been proposed by Kasprzyk (2011). On the basis of the function parameters, the author defined the date of maximum concentration and pollen season duration. This method is not only useful in the case of a unimodal curve of the pollen season, Kasprzyk and Walanus (2010) and Belmonte and Canela (2002) have fitted this type of model to multimodal pollen seasons.

Pollen grains and fungal spores can remain airborne for a long period of time after emission. The post-peak period is often longer than the pre-peak period, and the curve of the season is often characterised by positive skewness. As a result, gamma distribution-based models have been successfully used to describe the time sequence of pollen and fungal spores by Comtois (2000) and Belmonte and Canela (http://lap.uab.cat/aerobiologia/general/pdf/altres/MCC_XIIPC_slides.pdf; http://lap.uab.cat/aerobiologia/general/pdf/altres/TESAGamma.pdf), such models are successful in describing the seasonality of airborne pollen and fungal spores.

Another universal model has been presented by Ribeiro et al. (2007). It is a logistic model and its effectiveness does not depend on the duration or annual variation of the pollen season, or the taxa. By its nature, the model adapts itself to the cumulative curve of pollen concentration, as proposed by Pathirane (1975). The authors tested the model on several taxa, and it always produced an exceptionally good fit, almost 99 %. They stressed that the pattern followed by pollen seasons of various taxa from different regions can be quickly compared by the means of the function coefficients, in particular the growth rate coefficient. The models based on this function are often used to describe growth processes of living organisms (Gregorczyk 1991). A different approach was used by Stepalska and Wołek (2005). The period of occurrence of airborne *Alternaria* spores is exceptionally long and irregular. They divided it into two time intervals: the pre-peak and post-peak period, and both periods were fitted with a mathematical function that best described it. The best fitting was found for the exponential function, while the fitting for the power function was lower. Other models have been proposed that are based, among other things, on Poisson regression and Weibull distributions, gradient boosting method, transfer function model and autoregressive model (Limpert et al. 2008; Ocana-Peinado et al. 2008; Hilaire et al. 2012).

The main aim of this study was to develop mathematical models that explain dynamics in atmospheric concentrations of pollen and fungal spores. Plant taxa with different characteristics in the timing, duration and curve of their pollen seasons, as well as several fungal taxa, were selected for this analysis. Several models were examined, and their effectiveness in describing the occurrence of airborne pollen and fungal spores was compared. The effectiveness of the models was tested for on data collected at two separate sites differing in geobotanical and climatic conditions.

## Materials and methods

### Study area

Aerobiological monitoring was carried out in the centre of Rzeszów (N50°01′45″, E22°00′57″). Three years (2000–2002) were arbitrarily selected from several years of the database. Rzeszów is located in south-east Poland in the province of Carpathian Foothills. Mean annual precipitation is above 730 mm, and mean annual temperature is 8.8 °C. The warmest month is July (17.5 °C), and the coldest is January (−4.6 °C). The average vegetation period is 215–220 days. Rzeszów city boundaries have an agricultural character; the environs are a mosaic of forests and crop fields. The obtained results were compared with ones achieved in two other localities. The authors only possessed data from 1995.

- Zakopane (900 m a.s.l.) is a small town located in the Tatra Mountains, southern Poland. The mean annual precipitation ranges from 900 to 1,300 mm. The mean temperature in January is −4.9 °C and in July 14.7 °C. The average vegetation period is 180 days. In the close vicinity of Zakopane, there are more meadow areas than farmland. *Fagus silvatica*, *Abies alba* and *Picea excelsa* are an important component of forests.
- Ostrowiec Świętokrzyski (175 m a.s.l.) is a mid-sized city in Małopolska Upland in central Poland, where the mean annual precipitation is 550–650 mm and the vegetative period is 210 days. The mean temperature in January is −3.5 °C (the coldest month) and in July 18 °C (the warmest month). Vegetation in the town and its surroundings consists of ruderal vegetation, pine forests, semi-natural community of grasses and anthropomorphic habitats.

### Aerobiological monitoring

Investigations were continuously conducted at all sites using volumetric spore traps of the Hirst design (Hirst 1952). In Rzeszów, the trap was located about 12 m above the ground level, in Ostrowiec Św. 36 m and in Zakopane 10 m. Analyses by light microscope were carried out at magnification 400×. Pollen grains were counted along 12 vertical transects on each microscope slide. Each transect corresponds to a 2-h interval. The obtained result was expressed as the number of pollen grains in a cubic metre of air daily average (grains/m^3^) (Frenguelli 2003). Three pollen taxa with different characteristics in the timing, duration and curve of their pollen seasons were selected for analysis: *Artemisia, Betula,* *Rumex*. In addition, four fungal taxa were taken into consideration: *Cladosporium,* *Alternaria, Ganoderma* and *Botrytis*.

### Statistical analysis

#### Gaussian model

The bell curve fitting method [Gaussian curves, exp(−*x* ^2^)] was used to describe the seasons of airborne pollen and fungal spores.

$$y = f(t) = a \times \exp \left( { - 0.5 \times ((t - t_{1} )/s)^{2} } \right)$$

where *y*, daily count of pollen grains/fungal spores m^3^/24 h; *t*, day from the 1st January; *t*_1_, day of the modelled maximum pollen/spore concentration expressed as the number of days from the 1st January; *a*, the value of the modelled maximum pollen grains/spore concentration m^3^/24 h; and *s*, SD (in the sense of normal distribution) as a measure of half of duration of the maximum pollen/fungal spore season.

Using this function, a new method for defining the seasonal occurrence of airborne pollen and fungal spores was proposed. In the case of the Gaussian curve, 95.4 % of all values are theoretically included within the range of the *t*_*1*_ ± 2*s*. The dates calculated on this basis were compared with the dates based on the 95 % method (Goldberg et al. 1988), whereby the start of the season is the day when the cumulative sum of airborne pollen and fungal spores is 2.5 % of the total sum, and the end of the season is when this value reaches 97.5 %. This method for defining the limits of the season is frequently used in aerobiological studies (e.g. Stach et al. 2008).

Percentiles were also determined using the Excel formula for inverse normal distribution. Using the parameters determined on the basis of fitting this function, the occurrence of airborne pollen and fungal spores was presented as a cumulative concentration.

$$y = a \times ( 2 \times PI())^{0,5} \times s \times \text{NORMAL}.\;\text{DISTRIBUTION} \, \left( {t;t_{ 1} ;s;{\text{TRUTH}}} \right)$$

#### Gamma model

Gamma distribution that includes the right skewness of the curves representing the occurrence of pollen and fungal spores in the atmosphere.

$${\text{y}} = f\left( t \right) = a \times \gamma \left( {\left( {t - \, t_{\text{g}} } \right)/s;c} \right)$$

*a*, the value of the maximum pollen grain/fungal spore concentration per m^3^/24 h; *c*, shape parameter (the lower *c*, the larger skewness); *s*, scale parameter; *t*, number of days from the 1st January; and *t*_g_, shifting parameter for t_1_.

Day of the maximum pollen/fungal spore concentration can be expressed as a function of the above parameters:

$$t_{ 1} = \left[ {\left( {c - 1} \right) \times s} \right] + \, t_{\text{g}}$$

On the basis of this model, the dates of seasons of airborne pollen and fungal spores were defined using the inverse gamma distribution function in Excel.

#### Logistic differential model

The logistic function also has its differential version, which is less known. The formula is given below. The curve of the function can be normalised to the value of 1 as its maximal value (the same is necessary in the case of Gaussian distribution). In addition, if the horizontal axis (time axis) of the function will be normalised to obtain the same time space, e.g. 68 % quantiles, the two differential functions (the Gaussian and the logistic) produce very similar results. There are two different functions: the main difference between them is the way that the functions approaching zero. The Gaussian function reaches zero quickly, like the function exp(−*x* ^2^), while the logistic function is simply the exponential in that aspect; exp(−*x*). Whether this difference is essential for modelling pollen data is a question that needs to be answered experimentally. Comparing the results of fitting the integral and differential functions (to the integral and differential data) is not straightforward.

The formula of logistic differential function is:

$$y = f(t) = a \times 4 \times \text{exp}( - x)/(1 + \text{exp}(( - x))^{2}$$

where $x = \left( {t - t_{ 1} } \right)/{\text{s}}$ *t,* number of days from the 1st January; *t*_1_, day of the modelled maximum pollen/fungal spore concentration expressed as the number of days from the 1st January; *a*, the value of the modelled maximum pollen grains/fungal spores concentration m^3^/24 h; and *s*, SD as a measure of half of duration of the maximum pollen/fungal spore season.

#### Cumulative models: Gaussian and logistic

Time series of the pollen grains/fungal spores collected can be visualised/treated in two different ways. The first treatment is strictly connected to the method of data collection. The number of pollen grains/fungal spores is counted every day. That natural way can be called differential, since we do not simply have a number of items (pollen grains), but the number of items calculated (counted) per unit of time (day). That starts from zero on the 1st January and approaches a maximum somewhere in the summer (e.g.) and reverts to zero in December. Such behaviour in time is explained by, e.g. the bell-shaped curve (Gaussian probability distribution).

Another cumulative function frequently used for a wide variety of different purposes is a logistic one.

$${\text{y}} = f\left( x \right) = a/\left( { 1+ { \exp }\left( {b - c \times t} \right)} \right)$$

*a*, total sum of airborne pollen and fungal spores; *t*, number of days from the 1st January; *b*, position parameter; and *c*, scale parameter.

#### Rejection of outliers

**Fig. 1:**
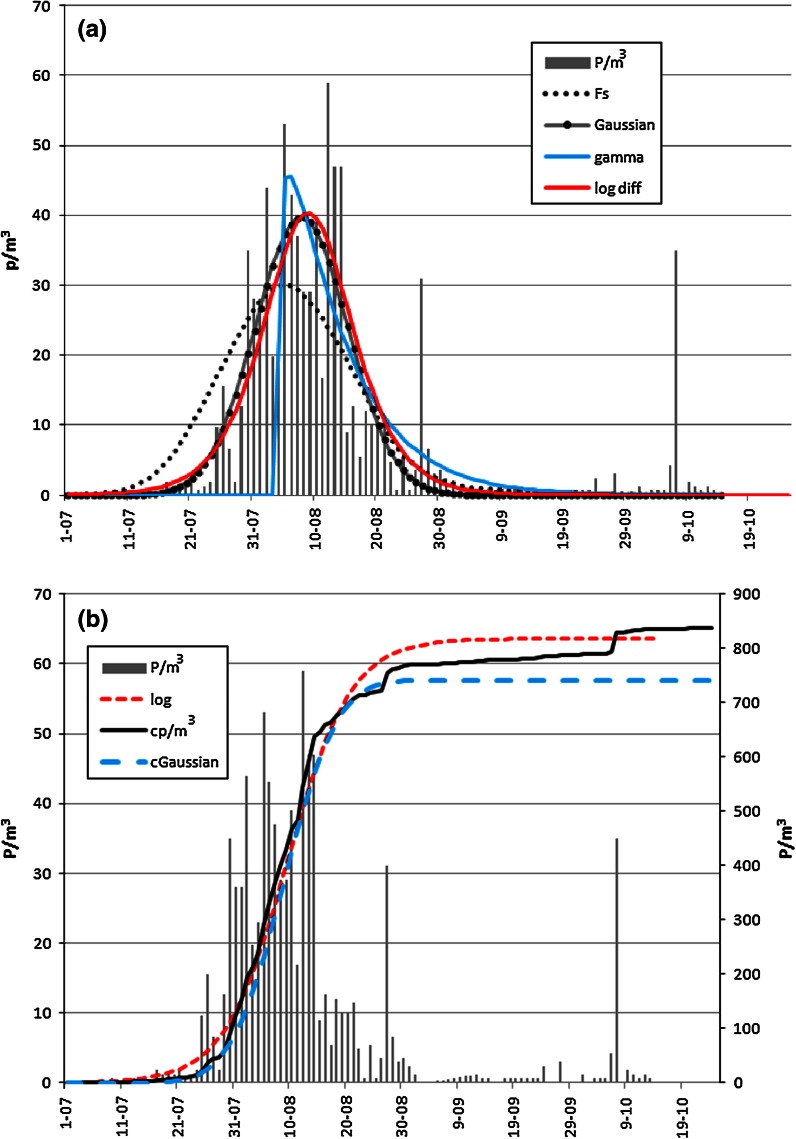
a Seasonal Artemisia pollen concentrations in Rzeszów in 2000 and adjusted curves of chosen models (Fs Friedman’s smoother). b Seasonal Artemisia pollen concentrations in Rzeszów in 2000 and adjusted cumulated curves of chosen models (cp cumulative pollen count)

Pollen and fungal spore data, like data of any origin, are not considered to be especially resistant to outliers. In the example (Fig. 1a), two outliers are evident in the post-peak period of the season. There is the possibility of simple gross errors, mistakes created when producing and processing the data. The most evident outlier in Fig. 1a is surrounded by days with low, but non-zero counts. Such points may be treated as outliers as well, depending on the given taxon and its behaviour of pollen season.

The models (not only Gaussian) can be considered robust where outliers far from the main season are concerned. This is because the function is close to zero in such a region. As a result, the sum of squares of differences is weekly dependent on small changes in the model parameters. A single outlier is too weak to move the whole model against the bulk of the ‘healthy’ data. As such, the simple idea of least squares works well with pollen data.

Nevertheless, evident outliers should be removed. We propose the simple rule, based on the ‘basement’ of the four surrounding days. The outlier (*x*_*i*_) should be rejected and replaced with the average value of neighbours when

$$x_{i} > {\text{ Sum}}/ 100\;{\text{and }}\;x_{i} > { 5} \times \left( {\left( {x_{i - 2} + \, x_{i + 2} } \right)/ 6+ \left( {x_{i - 1} + \, x_{i + 1} } \right)/ 3} \right)$$

where sum is a total, yearly sum of pollen grains/fungal spores.

**Fig. 2:**
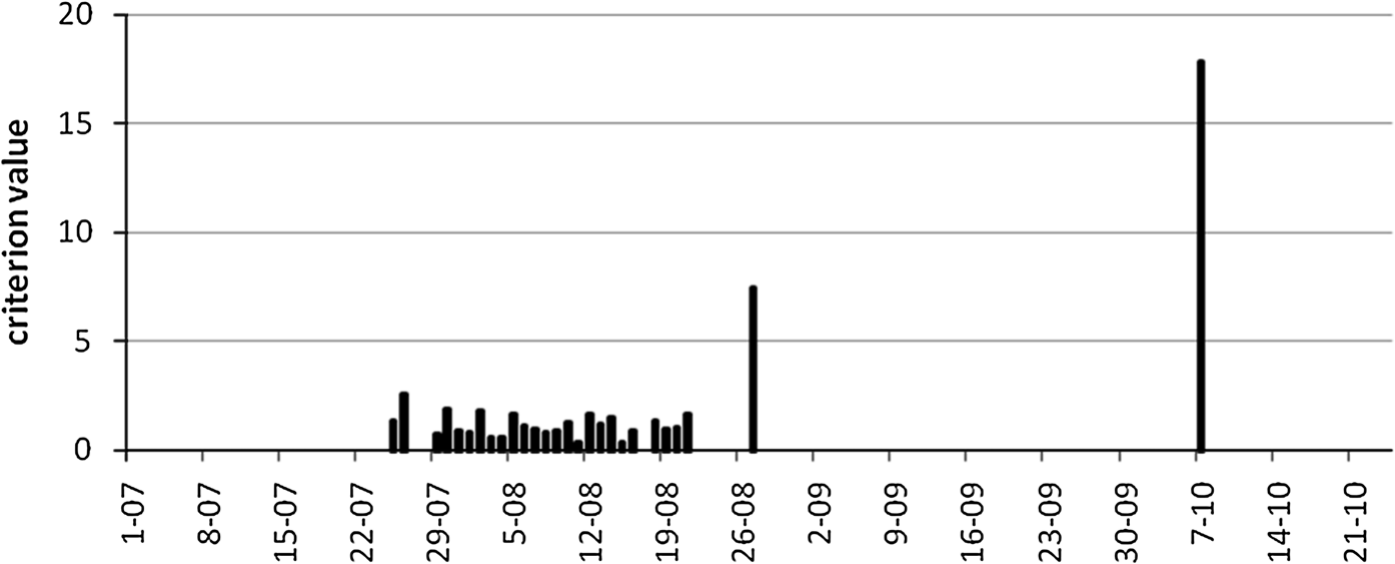
Operation of the criterion for rejection of outliers. Plotted are days only which confirm the criterion of share more than 1 % of the total sum. The plotted value (vertical axis) is that used in the criterion: $x_{i} /\left( {\left( {x_{i - 2} + x_{i + 2} } \right)/ 6 + \left( {x_{i - 1} + x_{i + 1} } \right)/ 3} \right)$. The value 5 seems to be good limiting one. The case of *Artemisia* concentrations in 2000 in Rzeszów is presented

The value of 5 in the formulae is chosen in an arbitrary way. The number 100 in the first part of the conjunction is arbitrary as well. However, it is of less influence. That part of the conjunction removes small counts from the suspicious range, what is necessary if the second, the main one part, of the conjunction is based on the relation to the surrounding days that may be empty of pollen (Fig. 2). One pollen grain surrounded by no pollen days cannot be treated as an outlier (1 > 5×0).

The so-called Friedman’s ‘super smoother’ was used to visualise time series of airborne pollen and fungal spores. It has been applied with statistical computing environment R (www.r-project.org). It is a ‘running lines’ smoother that chooses between three spans for the lines based on minimising cross-validated error.

The function ‘nonlinear estimation*’* Statistica 9.0 software was used for the analysis. The Levenberg–Marquardt method was applied to determine the initial values. The SE was given for all the function parameters. The goodness of fitting of the presented functions was expressed by the coefficient of determination. The statistical significance of the parameters of all functions was accepted at *α* ≤ 0.05.

## Results

### Models for pollen grains

In the case of the pollen taxa analysed, all the Gaussian function coefficients were statistically significant, but the goodness of fit of the function to the actual values varied significantly. The goodness of fit was also dependent on the type of taxa. The multi-species *Rumex* with medium or low concentrations of airborne pollen is characterised by poorly fitted functions. The Gaussian function was able to describe the occurrence of *Artemisia* pollen in the air in both Rzeszów and Ostrowiec Św. (Fig. 1a). The coefficient denoting the estimated maximum value showed the largest error. The other coefficients were characterised by lower SEs (Table 1).

**Table 1:** Parameters of Gaussian functions for chosen taxa

Pollen taxa	Year	*R* ^2^ (%)	*a* ± SE	*t*_1_ ± SE	*s* ± SE	Fungal taxa	Year	*R* ^2^ (%)	*a* ± SE	*t*_1_ ± SE	*s* ± SE
*Rumex*	2000	44	7.98 ± 0.47	156.0 ± 2.2	32.9 ± 2.2	*Alternaria*	2000	59	154 ± 7.0	190.80 ± 0.87	16.50 ± 0.87
*Rumex*	2001	53	12.58 ± 0.66	164.8 ± 1.4	22.6 ± 1.4	*Alternaria*	2001	59	115.4 ± 4.8	216.2 ± 1.8	37.0 ± 1.8
*Rumex*	2002	44	10.32 ± 0.63	151.1 ± 1.9	27.1 ± 1.9	*Alternaria*	2002	62	21 ± 10	227.96 ± 0.88	16.21 ± 0.88
*Rumex O*	1995	66	33.2 ± 1.3	174.0 ± 1.1	22.9 ± 1.1	*Alternaria O*	1995	65	220.7 ± 9.3	215.8 ± 1.2	24.3 ± 1.2
*Rumex Z*	1995	46	6.23 ± 0.38	174.9 ± 1.0	13.77 ± 0.98	*Alternaria Z*	1995	37	35.2 ± 2.6	213.7 ± 2.0	23.9 ± 2.0
*Artemisia*	2000	76	39.7 ± 1.3	221.60 ± 0.29	7.45 ± 0.29	*Botrytis*	2000	62	42.9 ± 1.9	195.1 ± 1.2	24.1 ± 1.2
*Artemisia* ^*a*^	2000	83	40.1 ± 1.1	221.54 ± 0.23	7.30 ± 0.23	*Botrytis*	2001	70	164.8 ± 5.9	203.04 ± 0.85	20.41 ± 0.85
*Artemisia*	2001	87	52.2 ± 1.2	218.70 ± 0.15	5.64 ± 0.15	*Botrytis*	2002	23	45.1 ± 3.7	204.0 ± 5.1	53.8 ± 3.2
*Artemisia*	2002	79	59.6 ± 1.9	221.05 ± 0.10	3.00 ± 0.11	*Cladosporium*	2000	62	4,410 ± 160	171.1 ± 1.5	36.9 ± 1.6
*Artemisia O*	1995	80	61.7 ± 1.9	215.79 ± 0.34	9.68 ± 0.34	*Cladosporium*	2001	82	6,940 ± 180	192.31 ± 0.71	23.75 ± 0.71
*Artemisia Z*	1995	37	7.87 ± 0.62	221.22 ± 0.84	9.27 ± 0.84	*Cladosporium*	2002	38	3,770 ± 220	204.3 ± 3.6	53.1 ± 3.7
*Betula*	2000	81	1,629 ± 49	112.40 ± 0.11	3.03 ± 0.11	*Cladosporium O*	1995	40	3,950 ± 240	224.4 ± 3.4	47.8 ± 3.5
*Betula*	2001	94	597.1 ± 9.5	119.243 ± 0.047	2.551 ± 0.047	*Cladosporium Z*	1995	57	5,050 ± 270	202.09 ± 0.27	4.44 ± 0.28
*Betula*	2002	52	79.2 ± 4.6	108.20 ± 0.50	7.34 ± 0.50	*Ganoderma*	2000	50	64.3 ± 2.7	211.4 ± 1.7	35.2 ± 1.7
*Betula O*	1995	88	551 ± 13	110.822 ± 0.022	0.813 ± 0.022	*Ganoderma*	2001	67	160.4 ± 5.8	244.8 ± 1.9	46.1 ± 2.1
*Betula Z*	1995	40	36.9 ± 2.7	122.26 ± 0.46	5.43 ± 0.46	*Ganoderma*	2002	70	426 ± 16	236.85 ± 0.78	17.91 ± 0.78

The formulae: $$y = f\left( t \right) = a \times { \exp } ( - 0. 5 \times \left( {\left( {t - t_{ 1} } \right)/s} \right)^{ 2} )$$

*R* ^2^ (%) determination coefficients, *SE* standard error of parameters of function, *Z* Zakopane, O Ostrowiec Świętokrzyski

^a^Without outliers

The function based on the Gaussian distribution appears to be resistant to outliers. This can be seen on the example of *Artemisia* in 2000. In that year, two jumps in concentrations were recorded after the main period of pollen release (Fig. 1a). These outliers would generally be expected that these outlying points would have some influence on the model, but this did not occur. Replacing outliers by the average of the surrounding days changes the values of the model and lowers the SE of estimation (Table 2).

**Table 2:** The relative SD (coefficient of variation) of four results obtained after removing outliers; the first, the second one, both and none (SE_out_)

	*a* (%)	*t*_1_ (%)	*s* (%)
SE_out_	0.5	0.02	1.2
SE_est_	3.4	0.13	4.0

The relative SE of estimation of the model parameters (SE_est_) is also given, for comparison. Since the SE_out_ is much less than SE_est_ the removing of outliers or not is simply irrelevant. Model is the Gauss function (*a*_1_ *, t*_1_ *, s* parameters of Gauss function)

The gamma function-based model shows the poorest fitting for pollen seasons. For many taxa, it was not possible to develop a model whose coefficients would be statistically significant (Table 6). As seen in Fig. 1a, the curve for *Artemisia* in 2000 only fits well in the post-peak period (the fitting for the pre-peak period was very poor). This is characteristic of all the taxa analysed. As in the case of the Gaussian function, a new model was prepared for *Artemisia* in 2000 after outliers were rejected. The goodness of fit of the model to the data was slightly better (it increased from 76 to 83 %), but the SEs for the function parameters were higher (Table 3).

**Table 3:** Parameters of gamma functions for chosen taxa (only statistically significant)

Pollen taxa	Year	*R* ^2^ (%)	*a* ± SE	*t*_*g*_ ± SE	*s* ± SE	*c* ± SE	Fungal taxa	Year	*R* ^2^ (%)	*a* ± SE	*t*_*g*_ ± SE	*s* ± SE	*c* ± SE
*Rumex*	2000	52	13.0 ± 1.6	124.999 ± 0.007	51.518 ± 7.747	1.049 ± 0.072	*Alternaria*	2001	65	186 ± 45	185 ± 9	59 ± 10	1.04 ± 0.11
*Rumex*	2001	55	40.6 ± 6.8	128.5 ± 3.3	18.399 ± 3.37	2.439 ± 0.503	*Alternaria*	2002	66	556 ± 48	209.998 ± 0.051	16.0 ± 1.7	1.59 ± 0.11
*Rumex*	2002	59	19.1 ± 2.1	127.99998 ± 0.006	35.562 ± 5.009	1.026 ± 0.068	*Alternaria* O	1995	67	360 ± 34	197.000 ± 0.004	35.9 ± 4.2	1.054 ± 0.058
*Rumex O*	1995	70	95.2 ± 9.8	141.6 ± 1.3	20.43 ± 2.383	1.981 ± 0.211	*Alternaria* Z	1995	46	68 ± 11	198.000 ± 0.006	28.0 ± 5.4	1.046 ± 0.097
*Rumex Z*	1995	49	18.1 ± 3.2	154.5 ± 2.0	13.152 ± 2.603	2.238 ± 0.462	*Botrytis*	2000	68	97 ± 9	168.74 ± 0.54	27.0 ± 3.1	1.47 ± 0.11
*Artemisia*	2000	57	69.1 ± 9.5	217.988 ± 0.044	8.39 ± 1.426	1.18 ± 0.120	*Botrytis*	2001	80	194 ± 47	191.0 ± 5.0	44.3 ± 9.5	1.06 ± 0.13
*Artemisia* ^a^	2000	62	77 ± 10	217.93 ± 0.13	7.164 ± 0.137	1.278 ± 0.135	*Botrytis*	2002	20	410 ± 160	206.4 ± 5.2	6.4 ± 2.2	3; NS
*Artemisia*	2001	60	93 ± 15	217.999 ± 0.010	4.895 ± 1.038	1.077 ± 0.154	*Cladosporium*	2001	64	11,460 ± 1,100	185.000 ± 0.005	27.3 ± 3.3	1.030 ± 0.060
*Artemisia*	2002	81	96.8 ± 9.6	219.0 ± 0.0022	4.612 ± 0.587	1.062 ± 0.095	*Cladosporium*	2002	22	8,910 ± 1,630	211.999 ± 0.022	28.2 ± 6.5	1.04 ± 0.11
*Artemisia O*	1995	19	440 ± 100	187.2 ± 5.9	3.438 ± 0.817	8.745 ± 3.608	*Cladosporium* O	1995	42	14,800 ± 4,100	142 ± 13	32 ± 10	3.0 ± 1.1
*Artemisia Z*	1995	54	21.89 ± 4.8	222.0 ± 0.0012	4.768 ± 1.419	1.055 ± 0.211	*Cladosporium* Z	1995	60	4,340 ± 710	197.0 ± 1.5	18.8 ± 6.8	0.70 ± 0.26
							*Ganoderma*	2000	62	80 ± 12	191.0 ± 2.8	64 ± 17	0.86 ± 0.13

The formulae $${\text{y}} = f\left( t \right) = a \times \gamma \left( {\left( {t - \, t_{\text{g}} } \right)/s;c} \right)$$

*R* ^2^ (%) determination coefficients, *SE* standard error of parameters, *Z* Zakopane, *O* Ostrowiec Świętokrzyski, *NS* not statistically significant estimates of the parameters

^a^Without outliers

The fitting of the differentiable logistic function produced good results, comparable with the Gaussian function (Tables 1, 4). In accordance with the principle adopted by the authors for *Artemisia* 2000, outliers were rejected and this model was refitted. The goodness of fit increased from 76 to 83 % (Table 4).

**Table 4:** Parameters of logistic differential function for chosen taxa

Pollen taxa	Year	*R* ^2^ (%)	*a* ± SE	*t*_1_ ± SE	*s* ± SE	Fungal taxa	Year	*R* ^2^ (%)	*a* ± SE	*t*_*1*_ ± SE	*s* ± SE
*Rumex*	2000	44	8.24 ± 0.50	154.3 ± 2.2	20.4 ± 1.5	*Alternaria*	2000	62	156.7 ± 6.8	191.07 ± 0.83	10.86 ± 0.58
*Rumex*	2001	53	12.83 ± 0.70	164.7 ± 1.4	14.4 ± 1.0	*Alternaria*	2001	63	122.7 ± 4.8	214.4 ± 1.5	22.1 ± 1.1
*Rumex*	2002	44	10.59 ± 0.67	149.1 ± 1.9	17.0 ± 1.3	*Alternaria*	2002	63	226 ± 10	227.41 ± 0.82	10.31 ± 0.58
*Rumex O*	1995	66	33.7 ± 1.4	173.7 ± 1.1	14.67 ± 0.78	*Alternaria O*	1995	70	225.7 ± 9.2	215.1 ± 1.1	15.46 ± 0.78
*Rumex Z*	1995	48	6.5 ± 0.40	174.57 ± 0.93	8.64 ± 0.67	*Alternaria Z*	1995	38	37.5 ± 2.7	212.0 ± 1.8	14.2 ± 1.2
*Artemisia*	2000	76	40.3 ± 1.4	221.68 ± 0.30	7.80 ± 0.22	*Botrytis*	2000	65	44.3 ± 1.9	194.2 ± 1.1	15.12 ± 0.79
*Artemisia* ^a^	2000	83	40.7 ± 1.2	221.58 ± 0.24	4.66 ± 0.17	*Botrytis*	2001	77	167.0 ± 5.7	202.86 ± 0.81	13.33 ± 0.57
*Artemisia*	2001	88	54.4 ± 1.3	218.67 ± 0.14	3.48 ± 0.10	*Botrytis*	2002	25	45.5 ± 3.7	205.5 ± 4.9	34.6 ± 3.5
*Artemisia*	2002	81	60.2 ± 1.9	221.01 ± 0.11	1.985 ± 0.077	*Cladosporium*	2000	67	4,580 ± 160	171.7 ± 1.4	22.9 ± 1.0
*Artemisia O*	1995	79	62.7 ± 2.0	215.73 ± 0.35	6.18 ± 0.24	*Cladosporium*	2001	84	7,240 ± 170	192.42 ± 0.61	14.74 ± 0.43
*Artemisia Z*	1995	37	8.39 ± 0.67	222.14 ± 0.78	5.43 ± 0.55	*Cladosporium*	2002	40	3,820 ± 220	205.5 ± 3.5	33.9 ± 2.4
*Betula*	2000	81	1,686.0 ± 5.3	112.59 ± 0.10	1.877 ± 0.072	*Cladosporium O*	1995	40	3,970 ± 240	223.4 ± 3.3	30.7 ± 2.3
*Betula*	2001	93	609 ± 10	119.259 ± 0.049	1.619 ± 0.034	*Cladosporium Z*	1995	58	5,120 ± 270	201.87 ± 0.26	2.88 ± 0.18
*Betula*	2002	53	82.5 ± 4.9	108.55 ± 0.48	4.54 ± 0.33	*Ganoderma*	2000	63	66.6 ± 2.7	210.2 ± 1.6	21.8 ± 1.1
*Betula O*	1995	89	554 ± 12	110.831 ± 0.023	0.540 ± 0.015	*Ganoderma*	2001	67	165.1 ± 5.9	245.6 ± 1.8	28.3 ± 1.3
*Betula Z*	1995	41	38.5 ± 2.9	122.53 ± 0.45	3.36 ± 0.31	*Ganoderma*	2002	71	431 ± 16	236.95 ± 0.76	11.69 ± 0.53

The formulae $$y = a \times 4 \times \text{exp}(( - x)/(1 + \text{exp}( - x))^{2} \;\text{where}\;x = (t - t_{1} )/s$$

*R* ^2^ (%) determination coefficients, *SE* standard error of parameters, Z Zakopane, *O* Ostrowiec Świętokrzyski

^a^Without outliers

The logistic function fitted the cumulative curves of pollen seasons of all the taxa analysed almost perfectly (Table 5; Fig. 1b). For *Artemisia,* the values of the parameter *c* were the highest and similar in successive years. This indicates a rapid increase in airborne pollen concentrations during the season. In the case of *Rumex*, the values of this parameter were the lowest, which indicates an entirely different pattern of the pollen season. There was a slow rate of increase in pollen grains of this taxon in each year of the study and at each site (Table 5).

**Table 5:** Parameters of logistic functions for chosen taxa

Pollen taxa	Year	*R* ^2^ (%)	*a* ± SE	*b* ± SE	*c* ± SE	Fungal taxa	Year	R ^2^ (%)	a ± SE	b ± SE	s ± SE
*Rumex*	2000	99.6	662.0 ± 1.7	8.13 ± 0.12	0.072 ± 0.001	*Alternaria*	2000	99.5	8,457 ± 24	9.41 ± 0.14	0.049 ± 0.00076
*Rumex*	2001	99.8	724.9 ± 1.2	12.09 ± 0.14	0.040 ± 0.001	*Alternaria*	2001	99.7	11,378 ± 31	8.910 ± 0.096	0.0406 ± 0.00046
*Rumex*	2002	99.5	715.4 ± 2.0	9.20 ± 0.17	0.058 ± 0.001	*Alternaria*	2002	99.7	10,360 ± 22	16.97 ± 0.22	0.0749 ± 0.00098
*Rumex* *O*	1995	99.8	1,857.7 ± 2.7	13.64 ± 0.15	0.078 ± 0.001	*Alternaria O*	1995	99.8	14,144 ± 30	13.60 ± 0.15	0.06219 ± 0.0007332
*Rumex* *Z*	1995	99.8	262.66 ± 0.47	14.36 ± 0.19	0.079 ± 0.001	*Alternaria Z*	1995	99.7	2,322.9 ± 5.7	12.56 ± 0.16	0.05785 ± 0.0007608
*Artemisia*	2000	99.8	817.5 ± 1.6	39.74 ± 0.80	0.178 ± 0.003	*Botrytis*	2000	99.7	2,469.4 ± 4.9	12.63 ± 0.15	0.06516 ± 0.000823
*Artemisia*	2001	99.9	784.87 ± 0.50	57.66 ± 0.47	0.263 ± 0.002	*Botrytis*	2001	99.2	10,316 ± 43	11.08 ± 0.22	0.0515 ± 0.00106
*Artemisia*	2002	99.7	624.3 ± 1.3	52.9 ± 1.3	0.237 ± 0.006	*Botrytis*	2002	99.3	6,106 ± 28	6.290 ± 0.094	0.031 ± 0.001
*Artemisia O*	1995	99.9	1,476.6 ± 1.1	38.82 ± 0.31	0.180 ± 0.001	*Cladosporium*	2000	99.7	419,560 ± 840	7.200 ± 0.071	0.04158 ± 0.000420
*Artemisia Z*	1995	99.8	181.37 ± 0.30	40.89 ± 0.73	0.186 ± 0.003	*Cladosporium*	2001	99.7	480,300 ± 1,030	10.00 ± 0.11	0.0511 ± 0.000587
*Betula*	2000	99.8	12,920.7 ± 6.6	56.98 ± 0.70	0.510 ± 0.006	*Cladosporium*	2002	99.6	499,100 ± 1,700	6.600 ± 0.073	0.032 ± 0.0004
*Betula*	2001	99.9	4,046.8 ± 1.5	69.20 ± 0.67	0.583 ± 0.006	*Cladosporium Z*	1995	99.5	108,570 ± 320	14.85 ± 0.27	0.0710 ± 0.001325
*Betula*	2002	99.7	1,600.0 ± 1.3	20.30 ± 0.25	0.190 ± 0.002	*Ganoderma*	2000	99.7	5,382 ± 12	9.376 ± 0.095	0.04447 ± 0.000464
*Betula O*	1995	99.7	1,821.375 ± 0.094	42.6 ± 1.1	0.382 ± 0.136	*Ganoderma*	2001	99.7	18,212 ± 67	9.17 ± 0.10	0.03772 ± 0.0004445
*Betula Z*	1995	99.5	747.7 ± 1.6	14.61 ± 0.34	0.115 ± 0.003	*Ganoderma*	2002	99.4	23,162 ± 85	13.38 ± 0.24	0.0580 ± 0.001060

The formulae $${\text{y}} = a/\left( { 1+ { \exp }\left( {b - c \times t} \right)} \right)$$

R^2^ (%) determination coefficients, *SE* standard error of parameters, *Z* Zakopane, *O* Ostrowiec Świętokrzyski

### Models for fungal spores

It was possible to fit the Gaussian distribution-based models to each type of spore in each year for all sites. The goodness of fit ranged from 23 % for *Botrytis* in 2002 to 82 % for *Cladosporium* in 2001. Although all the coefficients of the model were statistically significant, the highest error related to the coefficient *a*, which denotes the estimated maximum value. The errors of the coefficients relating to the peak date (*t*_*1*_) and to the parameter *s* were much lower (Table 1).

**Fig. 3:**
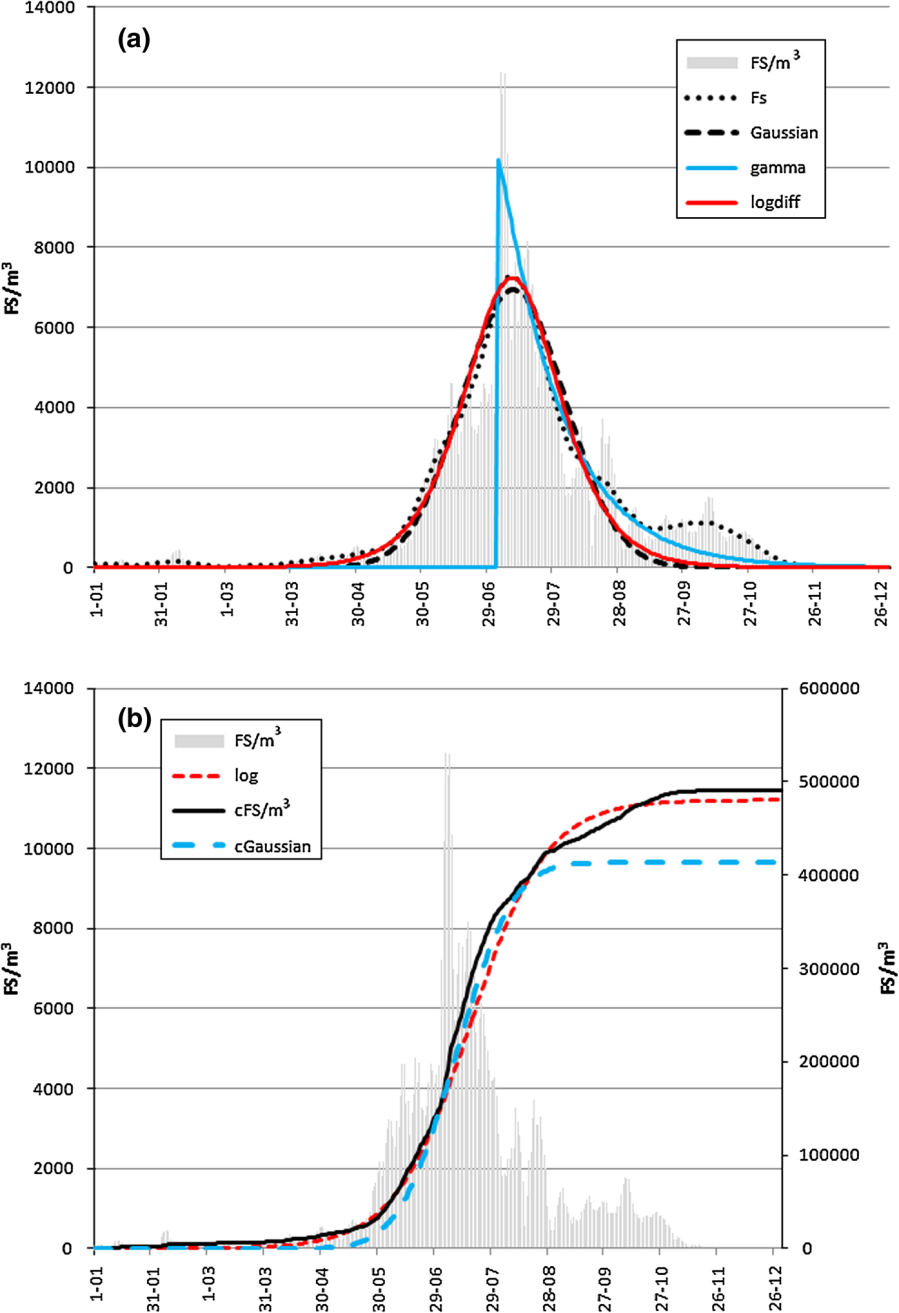
a Seasonal *Cladosporium* fungal spore concentrations in 2001 in Rzeszów and adjusted curves of chosen models (FS fungal spores; Fs Friedman’s smoother). b Seasonal *Cladosporium* fungal spore concentrations in 2001 in Rzeszów and adjusted cumulated curves of chosen models (cFS cumulative fungal spore count)

It was not possible to fit the gamma distribution-based model in all cases. In a few cases, the goodness of fit of the gamma distribution-based function was a slight improvement compared with the model based on the normal distribution. This function was the best at describing the seasonality of occurrence of airborne *Botrytis* spores in 2001, while the poorest fit was found also for *Botrytis* in the following year. In the case of *Cladosporium*, the curve of the gamma model exhibits a very good fit for only the post-peak period (Fig. 3a). The shape parameters indicating the skewness of the time series had similar results. For example, high values (>3) showed that the *Cladosporium* in Ostrowiec Świętokrzyski seasons had low skewness. Low values of these coefficients, e.g. *Cladosporium* in Zakopane and the *Ganoderma* season in 2000 in Rzeszów, indicate that these spores remained in the air for a long period of time at low concentrations (Table 3).

The models based on a differentiable logistic function showed a rather good fitting in many cases. The fitting was only below 50 % in four cases. The majority of *R* ^2^ coefficients were slightly higher than those for the Gaussian function, while the errors of the parameters *a* and *t*_*1*_ were slightly lower. This model fitted much better to the data in the case of *Ganoderma* in 2000, *Botrytis* in 2001 (Table 4).

The logistic function produced an almost perfect fit for the rate of increase in the number of airborne fungal spores in all cases (Fig. 3b). However, parameter *a*, which denotes the cumulative total of spores, had a large or very large error. The values of parameter *c* are generally very low, which shows that the rate of increase in concentrations was also very low (Table 5). The logistic model curve roughly fitted the cumulative curve for *Cladosporium* spores in 2001 (Fig. 3b).

### Graphical presentation of seasons of airborne pollen and fungal spores

All presented methods smooth the diagrams presenting the occurrence of airborne pollen or fungal spores to a greater or lesser degree. After visual analysis of Figs. 1a, 3a, we state that the Friedman’s ‘super smoother’ method is very good. It described the unimodal season as well as multimodal seasons like *Cladosporium* in 2001.

### Calculation of dates of pollen/fungal spore seasons

The use of a Gaussian function smooths the curves in airborne pollen and fungal spore concentrations. Sporadic occurrences of airborne pollen and fungal spores, i.e. days when pollen or fungal spores were recorded before or after the period of continuous occurrence, were automatically rejected from the model. For example, there were two episodes recorded in 2000 when airborne *Artemisia* pollen increased for just 1 day; at the end of August and at the beginning of October. Pollen seasons defined using the Gaussian and gamma functions failed to capture these peaks. The models determined the end of the season to be: (1) Gaussian function—end of August; (2) gamma function—beginning of September. According to the traditional percentage method, the season ended at the beginning of October (when 97.5 % had been reached) and both peaks were included. The two methods for defining pollen seasons based on the Gaussian function were then compared. The pollen season defined using percentiles from the modelled data was always shorter than the one defined using the method *t*_*1*_ ± 2*s*. The differences in end date were either very small or there were no differences, for instance for *Artemisia* in 2000 and 2002 as well as for *Alternaria* in 2002. Much larger differences were found in the case of start date—the dates based on percentiles were always earlier than for the method *t*_*1*_ ± 2*s* (Table 6).

**Table 6:** Dates and duration of pollen/fungal spores seasons calculated according to percentage method and three models based on Gaussian and gamma distributions

Taxa		Start of the season				
		*Alternaria*		*Artemisia*		
Years		2001	2002	2000	2001	2002
Percentage method	Quantiles 2.5 and 97.5 %	04.05	25.05	26.07	26.07	26.07
Gaussian	t_1_ ± 2s	21.05	14.07	26.07	25.07	02.08
Gauss	Quantiles 2.5 and 97.5 %	09.07	04.08	03.08	31.07	06.08
gamma	Quantiles 2.5 and 97.5 %	22.07	07.08	05.08	05.08	06.08

Taxa		End of the season				
		*Alternaria*		*Artemisia*		
Years		2001	2002	2000	2001	2002
Percentage method	Quantiles 2.5 and 97.5 %	27.10	13.10	08.10	01.09	08.09
Gaussian	t_1_ ± 2s	16.10	16.09	23.08	18.08	14.08
Gauss	Quantiles 2.5 and 97.5 %	15.10	16.09	23.08	17.08	14.08
gamma	Quantiles 2.5 and 97.5 %	13.02^a^	11.10	08.09	24.08	24.08

Taxa		Lenght of the season				
		*Alternaria*		*Artemisia*		
Years		2001	2002	2000	2001	2002
Percentage method	Quantiles 2.5 and 97.5 %	177	142	74	38	45
Gaussian	t_1_ ± 2s	149	66	29	25	13
Gauss	Quantiles 2.5 and 97.5 %	99	44	21	18	9
gamma	Quantiles 2.5 and 97.5 %	207	66	35	20	19

*t*_1_ Estimated day of maximum concentration

^a^Next year

## Discussion

Aerobiological data are an example of data that do not have a normal distribution (Comtois 2000). They are characterised by positive skewness, and different data transformations (finding the logarithm, extraction of roots) do not change their basic shape. This limits the range of methods that scientists can use to analyse these data (Stępalska and Wołek 2005; Fernandez-Llamares et al. 2013). However, if we illustrate the occurrence of airborne pollen and fungal spores in time, it can be seen that the curves are close to being symmetrical and it is even clearer after Fisher’s smoothing method is applied (Belmonte and Canela 2002; Hilaire et al. 2012). A question therefore arises whether a good model for defining seasons of occurrence of airborne pollen and fungal spores can be prepared based on the Gaussian distribution and the differentiable logistic function (unimodal functions). According to the authors’ knowledge, the latter function has not previously been used for this type of data.

The Gaussian and differential logistic models shown in this paper were very good at describing pollen seasons with just one peak. These are typical for pollen types with just one dominant species in the flora and when the weather, in particular temperature, is stable during the pollination period. Such types of season are characteristic of *Artemisia*. According to a floristic study conducted in Rzeszow by Święs (1993), the most frequently found mugwort species is *A. vulgaris*. Modified Gaussian and differential logistic models can produce a good fit for multimodal pollen seasons in which concentrations are relatively high and the peaks are clearly defined. Such a model for the Gaussian function was presented by Kasprzyk and Walanus (2010) for a bimodal pattern of the grass pollen season where it performed well (on average with 70 % explanation). Belmonte and Canela (2002) successfully used this model for the multimodal Urticaceae season. The Gaussian and differential logistic functions poorly describe pollen seasons of taxa, which have: pollen that remains in the air for a long time; low levels of pollen in the air; and peaks that are not particularly evident. Such seasons are characteristic of the multi-species genus *Rumex* (Piotrowska 2012). The functions do not fit the data particularly well, generally at a level lower than 50 %. Such a result is not satisfactory. *Alternaria* and *Cladosporium* spore concentrations are high or very high and the peaks are often clearly defined, but the Gaussian and differential models often failed to fit the data. The seasonal pattern is strongly affected by external factors, such as the availability of substrates and weather conditions, as well as agronomic treatments that could alter the amount of fungal spore in the air (Corden et al. 2003; Skjøth et al. 2012). The goodness of fit is not dependent on where research is carried out, but the behaviour of the pollen season and type of taxa.

The curves representing the occurrence of pollen grains of some taxa are often characterised by a longer post-peak period (Wołek and Myszkowska 2008; Myszkowska et al. 2011; Dahl et al. 2013; Fernandez-Llamares et al. 2013). Grewling et al. (2012) report that although *Betula* pollen seasons can last more than 1 month, 85 % of birch pollen appears within the first 2 weeks, and if the temperature reaches 20 °C, it is 90 % within the first few days. Myszkowska and Piotrowicz (2009) state that *Betula* pollen concentrations are lower and the pollen seasons are longer when there are unstable weather conditions during the period preceding pollen release; in such cases an increase in pollen concentration was often recorded at the end of the season. The analysis of our results showed a similar pattern of the seasons of some taxa. Therefore, an attempt was made to develop a model based on the gamma distribution, which should incorporate these features. Belmonte and Canela indicated that this was a good method both for pollen and fungal spore seasons (http://lap.uab.cat/aerobiologia/general/pdf/altres/TESAGamma.pdf; http://lap.uab.cat/aerobiologia/general/pdf/altres/MCC_XIIPC_slides.pdf). The presented results do not confirm this. The models have a poor fit or they are not statistically significant. Despite the fact that season dates can be defined on the basis of the function parameters, it seems that they do not correspond to reality. The beginning of the season is defined too late. In the case of *Artemisia*, this date only differs from the peak date by 3 days.

Different methods presenting cumulative data are given in the literature. The cumulative version of the distribution function visualises the main feature of the time series, i.e. the maximum of the season, in a rather hidden way. Cumulative curves are S-shaped and such a curve can be produced for the growth of organisms or, as proposed by Pathirane (1975) and Ribeiro et al. (2007), for the pattern of airborne pollen concentrations. They can be described using models based on the logistic function, and its basic formula and numerous modifications have been presented, among others, by Gregorczyk (1991) for plant growth. The presented results confirm the statement of Ribeiro et al. (2007) that the model performs well and the strength of the fitting is not dependent on the type of taxon. Compared with logistic functions, the cumulative Gaussian distribution performed poorly.

Outlying or suspicious values (a mistake in the identification of pollen grains, equipment error) can reduce the effectiveness of models. Decisions about the rejection of atypical or suspicious results should be made on a case by case basis, taking into account all possible factors affecting the pattern of occurrence of airborne pollen and fungal spores such as: taxon, the specificity of pollination or sporulation, the flora of the region, human activity, or the purpose of the research.

Several methods for defining the limits of the pollen season are given in the literature. Jato et al. (2006) compared them in detail and proposed that the type of taxon (mono- or multi-species) and sensitivity to weather changes should be taken into account in defining pollen season dates. We have defined the seasonal occurrence of airborne pollen and fungal spores by making an assumption that they are regular and close to symmetry. We have based the definitions on the Gaussian distribution what is novelty in aerobiological study. The dates of seasons can be very quickly calculated having only two parameters: the estimated time of maximum and SD (*t*_*1*_ ± *s*). The proposed method should be used when we are interested in a condensed season. The second method is based on percentiles calculated from adjusted results, and it is more time-consuming. The analysis of the results shows that the type of method should be adjusted to the type of taxa, which is consistent with Jato et al. (2006) suggestions.

## Conclusions

The models based on the Gaussian and differentiated logistic functions are robust and suitable for pollen grains and fungal spores. They are recommended for unimodal seasons. They are quite resistant to outliers. Two novel methods for defining pollen seasons have been proposed for aerobiological studies. They are based on the Gaussian function. We suggest that they should be used to define the main pollen season. Comparisons of season dates defined by several methods show once again that, depending on the purpose of the research, the use of the same method for all taxa is not always justified and; moreover, one should consider whether in some cases the start and end of the season should be defined using different methods. In spite of the fact that seasonal curves are often characterised by positive skewness, the model based on the gamma distribution was not very effective. Friedman’s smoother method is very good for graphically presentation of time series of pollen and fungal spores. It is not easy method; it needs the knowledge of R programme language.
